# Navigating Early Alzheimer's Diagnosis: A Comprehensive Review of Diagnostic Innovations

**DOI:** 10.7759/cureus.44937

**Published:** 2023-09-09

**Authors:** Anup Juganavar, Abhishek Joshi, Tejas Shegekar

**Affiliations:** 1 Medicine, Jawaharlal Nehru Medical College, Datta Meghe Institute of Higher Education and Research, Wardha, IND; 2 Community Medicine, Jawaharlal Nehru Medical College, Datta Meghe Institute of Higher Education and Research, Wardha, IND

**Keywords:** biomarkers, diagnostic tools, machine learning technique, neuroimaging techniques, lipid biopsy, alzheimer's disease

## Abstract

The hunt for early Alzheimer's disease detection has created cutting-edge diagnostic instruments with enormous promise. This article examines the many facets of these developments, focusing on how they have revolutionised diagnosis and patient outcomes. These tools make it possible to detect tiny brain changes even before they give birth to clinical symptoms by combining cutting-edge biomarkers, neuroimaging methods, and machine-learning algorithms. A significant opportunity for therapies that can slow the course of the disease exists during this early detection stage. Additionally, these cutting-edge techniques improve diagnostic precision, objectivity, and accessibility. Liquid biopsies and blood-based biomarkers provide non-invasive alternatives, filling accessibility gaps in diagnosis. While issues with standardisation, ethics, and data integration continue, collaboration within research, clinical practice, and policy realms fuels positive developments. As technology advances, the way towards better Alzheimer's diagnosis becomes more evident, giving patients and families dealing with this difficult illness fresh hope. The synergy between scientific advancement and compassionate treatment is crucial for improving Alzheimer's disease diagnosis, as this paper emphasises.

## Introduction and background

Alzheimer's is a common neurodegenerative condition marked by a slow loss of cognitive function and memory. It accounts for 60-80% of dementia cases, making it the most likely cause of dementia. The disease typically affects older persons, with the risk dramatically rising beyond 65 years [1]. Amyloid plaques and neurofibrillary tangles build up in the brain in Alzheimer's disease, resulting in the death of neurons and malfunctioning synapses [2]. Cognitive deficiencies, behavioural issues, and functional impairment brought on by these pathological changes significantly negatively influence the quality of life of the affected person and their carers.

The importance of early Alzheimer's disease identification cannot be overstated for several convincing reasons. To start with, early detection enables sufferers and their families to make plans and get ready for the future. It allows one to make thoughtful choices about financial, legal, and medical issues, ensuring the necessary support systems are in place. Second, early identification makes it easier to obtain various therapies and interventions that might halt the spread of the illness or lessen symptoms. Even though there is currently no cure for Alzheimer's, several drugs and treatment techniques can help control symptoms and improve general well-being. Additionally, early diagnosis makes it possible for people to participate in clinical trials and research projects meant to provide cutting-edge cures or preventative measures. People in the early stages of the disease are frequently needed for this research, and their involvement can significantly advance scientific knowledge and lead to treatment advances [1].

To diagnose Alzheimer's disease early, diagnostic techniques are essential. They help medical practitioners identify cognitive impairment, differentiate Alzheimer's disease from other types of dementia, and track the course of the disease. Creating and using trustworthy diagnostic tools is essential for increasing diagnostic precision, decreasing misdiagnosis rates, and permitting prompt action. The creation of diagnostic techniques for Alzheimer's disease has advanced significantly over time. Traditional approaches have been the cornerstone of diagnosis, including clinical examination, medical history assessment, cognitive testing, and brain imaging techniques [2]. These techniques have drawbacks, such as late-stage findings, subjectivity, and invasive methods. Recent developments in neuroimaging methods, biomarkers, and machine learning algorithms have created novel opportunities for early detection and increased diagnostic precision [3]. The emergence of the newest diagnostic techniques has the potential to transform the process of early detection of Alzheimer's disease completely. It might revolutionize clinical practice, improve patient care, and accelerate the development of new treatment procedures and preventative measures.

## Review

Methodology

**Figure 1:**
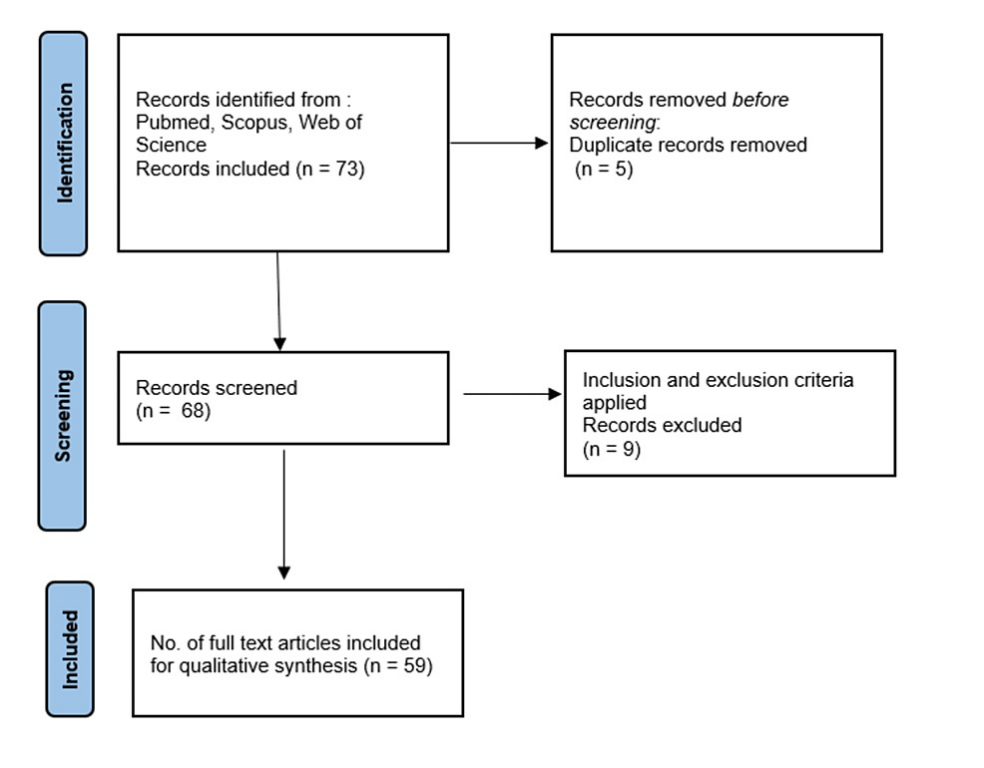
The PRISMA (Preferred Reporting Items for Systematic Reviews and Meta-Analyses) flow diagram illustrating the process of study selection

To ensure a comprehensive exploration of diagnostic tools for early Alzheimer's disease detection, a systematic search was executed. The search was conducted in prominent databases, including PubMed, Scopus, and Web of Science, on 19 July 2023. The search utilised relevant keywords and Medical Subject Headings (MeSH) terms encompassing Alzheimer's disease, early detection, diagnostic tools, biomarkers, neuroimaging, and machine learning. The study selection adhered to predetermined criteria, focusing on studies directly related to innovative diagnostic tools for early Alzheimer's detection. Non-English studies, non-peer-reviewed articles, and those focused primarily on other neurodegenerative disorders were excluded. The initial search yielded 73 articles, and after removing duplicates, a two-phase screening process led to 68 articles for full-text review. Ultimately, 59 studies were incorporated into the final review. For visual clarity, the PRISMA (Preferred Reporting Items for Systematic Reviews and Meta-Analyses) flow diagram (Figure 1) below succinctly depicts the article selection process.

Traditional diagnostic methods for Alzheimer's disease

Clinical Evaluation and Medical History

Examining the patient's clinical condition and medical history are essential steps in diagnosing Alzheimer's disease. To learn about the patient's complaints, history of illness, and cognitive impairment, healthcare experts interview patients and their carers in-depth [4]. They evaluate several things, including behavioural and functional limitations and changes in memory, language, thinking, and problem-solving skills [5]. During the clinical examination, doctors may use standardised instruments to evaluate cognitive function and review for dementia, such as the Mini-Mental State Examination (MMSE) or the Montreal Cognitive Assessment (MoCA) [6]. These assessments gauge a patient's cognitive ability by measuring their memory, attention, language, and visuospatial skills. During the medical history evaluation, details on the patient's current and historical health issues, usage of medications, and family history of dementia are gathered [7]. A thorough medical history is necessary for a definitive diagnosis of Alzheimer's disease since other medical disorders and drugs can cause cognitive impairment or mimic its symptoms [8].

Brain Imaging Techniques

Techniques for imaging the brain are essential in diagnosing Alzheimer's disease. They help identify important disease-related biomarkers by offering insightful information on the structural and functional alterations happening in the brain [9].

An in-depth brain anatomy image is produced using the non-invasive imaging method known as magnetic resonance imaging (MRI). Healthcare practitioners may analyse brain shrinkage, spot anomalies, and spot structural changes linked to Alzheimer's disease because of the high-resolution pictures it gives [10]. An Alzheimer's diagnosis can be supported by MRI results, which may help rule out alternative causes of cognitive impairment. The characteristic pathology of Alzheimer's disease, i.e., beta-amyloid plaques and neurofibrillary tangles, may be seen with PET scans [11]. Numerous PET tracers have been designed, including florbetapir, flutemetamol, and florbetaben, to visualise and measure the amount of beta-amyloid plaques in the brain, assisting in the identification and differential diagnosis of Alzheimer's disease [12].

Single-photon emission computed tomography (SPECT) imaging can reveal essential details regarding regional brain function and illustrate regions with decreased blood flow or aberrant metabolism [13]. In addition to assisting in the early diagnosis and tracking of the illness's course, SPECT scans can distinguish Alzheimer's disease from other forms of dementia. An understanding of brain activity and functional connectivity may be gained by functional magnetic resonance imaging (fMRI), which tracks changes in cerebral blood flow and oxygenation levels in the brain. It can assist in locating anomalies in neural networks and evaluating the functional limitations brought on by Alzheimer's disease [14]. Understanding illness processes and cognitive decline, fMRI is especially helpful in examining the brain's functional alterations during cognitive activities and resting-state situations.

Cognitive Assessments

Diagnosing Alzheimer's disease and determining the degree of cognitive impairment depend heavily on cognitive exams. These tests offer quantifiable measurements of cognitive performance in various areas, assisting in identifying particular impairments and monitoring long-term improvements [15].

MMSE is a frequently employed cognitive screening measure that evaluates several cognitive domains, including orientation, attention, memory, language, and visual-spatial abilities [6]. A sequence of questions and exercises, including word recall, counting backwards, and obeying instructions, make up the test. A total score between 0 and 30 is possible; lower values denote more severe cognitive impairment. Compared to the MMSE, the MoCA is another often-used cognitive screening instrument offering a more thorough cognitive function assessment. Additionally, it evaluates executive skills, visuospatial skills, and attention to detail [16]. Higher overall scores, which range from 0 to 30, indicate superior cognitive function.

A more thorough cognitive evaluation instrument created especially for Alzheimer's disease clinical trials is the Alzheimer's disease assessment scale-cognitive subscale (ADAS-Cog). It assesses several areas of cognition, such as memory, language, praxis, and orientation [17]. The ADAS-Cog comprises several tasks and questions; depending on the version taken, there are different score ranges. The clinical dementia rating (CDR) is a thorough evaluation instrument that rates cognitive and functional abilities across several categories. It comprises interviews with the patient and an informant (such as a family member or carer) and assigns a score of 0-5 to cognitive impairment. The CDR assists in assessing the degree of dementia and offers a comprehensive evaluation of cognitive function [18].

Limitations of traditional diagnostic methods

Late-Stage Detection

Conventional diagnostic techniques for Alzheimer's disease tend to identify the condition only after severe neurodegeneration and cognitive impairment, which is one of its significant drawbacks. A diagnosis can be delayed due to relying too heavily on clinical assessments, medical history evaluations, and cognitive testing, missing out on possibilities for early intervention and treatment [19]. The treatment of the disease and the potential advantages of therapeutic approaches are complicated by late-stage identification. The brain may already be irreparably damaged by the time symptoms emerge and a diagnosis is obtained. Early identification is essential because it enables the application of prompt therapies and tactics to slow the course of illness and enhance patient outcomes [3].

Subjectivity and Variability

The inherent subjectivity and inconsistency in the comprehension of clinical evaluations and cognitive tests is another drawback of conventional diagnostic techniques for Alzheimer's disease. These techniques rely significantly on the professional opinion and knowledge of medical experts, which raises the possibility of inter-rater variability and subjective biases [20]. Patient's symptoms and functional limitations are prone to subjective judgements during clinical examinations like interviews and observations. Due to healthcare practitioners' diverse degrees of knowledge and interpretative abilities, conflicting diagnoses and treatment suggestions may result [21]. Particularly in its early stages, when symptoms may be modest, the subjectivity and unpredictability of conventional diagnostic techniques can make it difficult to accurately and reliably diagnose Alzheimer's disease. It highlights the requirement for more impartial, quantitative diagnostic instruments that can deliver reliable outcomes.

Expensive and Invasive Procedures

Conventional diagnostic techniques for Alzheimer's disease sometimes entail pricy, intrusive procedures that might be difficult to access, costly, and unpopular with patients. The brain imaging techniques for Alzheimer's include MRI, PET, and SPECT. However, these methods may be expensive and require specialised tools and skilled staff [10-13,22]. Additionally, introducing radioactive tracers may be necessary for some imaging procedures, which can be hazardous and logistically challenging. A thorough evaluation of cognitive function may be obtained by neuropsychological testing; however, doing so often takes time, knowledge, and specialised tools. These tests are expensive and less available in some healthcare settings since they frequently require neuropsychologists or other specialised professionals for administration and interpretation [23]. When it comes to the existence of specific biomarkers linked to Alzheimer's disease, including beta-amyloid and tau proteins, CSF examination can offer helpful information. However, getting CSF samples necessitates lumbar puncture, an intrusive procedure with potential dangers [24]. Furthermore, most CSF analyses are carried out in specialised labs, raising costs and logistical difficulties.

Advancements in diagnostic tools for early detection

Biomarkers and Cerebrospinal Fluid Analysis

The use of biomarkers, notably through CSF examination, has been a focus of recent developments in diagnostic methods for the early diagnosis of Alzheimer's disease. These biomarkers allow for the identification of those at risk or who are just beginning to develop the disease, and they also offer important insights into the underlying pathological processes [25]. The proteins tau and beta-amyloid are important indicators linked to Alzheimer's disease. The disease's distinguishing characteristics include aberrant tau protein phosphorylation and increased amounts of beta-amyloid in the brain [26]. The measurement of beta-amyloid (Aβ42), total tau (t-tau), and phosphorylated tau (p-tau) proteins in CSF might provide critical diagnostic data [27]. The usefulness of CSF biomarkers in distinguishing people with Alzheimer's disease from healthy controls and those with other kinds of dementia has been shown in several studies [28]. Alzheimer's pathology has been linked to decreased levels of Aβ42 and elevated levels of t-tau and p-tau in the CSF, which may be beneficial in the early identification of the disease [29]. More biomarkers are being researched to diagnose Alzheimer's disease early. Among these are indicators of vascular abnormalities, synaptic dysfunction, neurodegeneration, and neuroinflammatory markers [30]. For instance, the neurofilament light chain (NfL) has demonstrated potential as a marker of neurodegeneration and the development of illness [31].

Targeted Positron Emission Tomography (PET) Imaging

A sophisticated diagnostic method that has shown tremendous promise in the early diagnosis of Alzheimer's disease is PET imaging. It enables the visualisation and measurement of specific molecular processes in the brain, revealing important details about the disease's underlying pathophysiology [32]. For the early identification of beta-amyloid plaques, one of the indicators of Alzheimer's disease, amyloid PET imaging is beneficial. In the brain, beta-amyloid aggregates are mainly bound by radiotracers such as Pittsburgh compound B (PiB), florbetapir, florbetaben, and flutemetamol [33]. Amyloid PET imaging aids in the early detection of patients with Alzheimer's disease by displaying the location and buildup of beta-amyloid. According to studies, amyloid PET imaging can help differentiate between people with Alzheimer's disease and people with other kinds of dementia or healthy controls [34]. It has shown great sensitivity and specificity in identifying beta-amyloid deposition, allowing for precise diagnosis and better patient treatment [35]. Tau PET imaging is another emerging application of PET technology in Alzheimer's disease diagnosis. Radiotracers such as 18F-AV-1451 (also known as T807 or flortaucipir) and 18F-MK-6240 selectively bind to tau protein aggregates, which are associated with neurofibrillary tangles in the brain. Tau pathology may be seen and quantified by tau PET imaging, which adds to our understanding of the course and severity of the illness. Alzheimer's disease can be distinguished from other neurodegenerative conditions using tau PET imaging, which has shown promise in identifying tau damage in people [36].

Blood-Based Biomarkers and Liquid Biopsies

The creation of liquid biopsies and blood-based biomarkers as non-invasive diagnostic methods for the early diagnosis of Alzheimer's disease has garnered growing interest in recent years. When compared to invasive treatments like CSF analysis or neuroimaging techniques, these methods have the benefit of being easily accessible and perhaps being less expensive.

There have been studies on several putative blood-based biomarkers. A number of amyloid-beta (Aβ) peptides, notably Aβ42 and Aβ40, have been investigated as blood-based biomarkers for Alzheimer's disease [37]. Changes in the ratios or concentrations of these peptides in the blood may indicate an underlying amyloid condition. The production of neurofibrillary tangles in Alzheimer's disease is thought to be influenced by tau proteins, particularly phosphorylated versions (p-tau). Total tau or p-tau blood-based tests have demonstrated potential in identifying Alzheimer's disease [38]. When neurons are damaged or degenerate, a protein called NfL that is present in neurons is released into the circulation. Increased NfL levels have been linked to neurodegenerative conditions such as Alzheimer's disease [39]. Blood-based NfL tests are a possible indicator of illness advancement and therapeutic response.

Biomarkers in bodily fluids such as blood, saliva, urine, and CSF are analysed during liquid biopsies. They provide a simple, accessible method for identifying the molecular alterations linked to Alzheimer's disease. Blood samples with circulating cell-free DNA or RNA can be examined to learn about genetic or epigenetic changes particular to a specific illness [40]. Potential biomarkers for Alzheimer's may include changes in certain genes or gene expression patterns. Exosomes may be separated from different bodily fluids and then examined for disease-specific indicators. Exosomal biomarkers have demonstrated potential in the early diagnosis and follow-up of neurodegenerative illnesses, including Alzheimer's disease [41].

Neuroimaging Techniques and Machine Learning Algorithms

Machine learning algorithms paired with neuroimaging methods have become effective tools for the early identification and diagnosis of Alzheimer's disease. These methods use neuroimaging's capacity to record structural, functional, and chemical changes in the brain. At the same time, machine learning techniques make it possible to analyse and comprehend intricate patterns in imaging data. An understanding of functional brain activity may be gained by fMRI, which tracks variations in blood oxygenation levels in the brain. It is able to spot changes in connectivity and activity patterns linked to Alzheimer's disease [42]. Complex functional connectivity networks may be analysed using machine learning algorithms applied to fMRI data to find biomarkers that distinguish people with Alzheimer's disease from healthy controls. For instance, early functional connectivity abnormalities in Alzheimer's disease have shown promise when detected using graph-based methods in conjunction with support vector machine classifiers [43].

Benefits of new diagnostic tools

Early Identification of Alzheimer's Disease

There are several advantages to developing new diagnostic methods for the early diagnosis of Alzheimer's disease. Early detection enables prompt intervention and therapy, which may halt the course of the disease and enhance patient outcomes. Identifying small changes in the brain may be done using biomarkers, neuroimaging, and machine-learning techniques even before clinical symptoms appear [44]. This early detection helps to focus therapies at the prodromal or preclinical periods, when they may be most successful.

Improved Accuracy and Objectivity

The new diagnostic tools use cutting-edge technology and quantitative techniques, improving the objectivity and accuracy of Alzheimer's disease diagnosis. Combining machine learning algorithms with biomarkers like beta-amyloid and tau in blood or CSF enhances the accuracy of diagnosis [45]. This lessens the dependence on arbitrary clinical judgements, reducing the variation in diagnosis across various healthcare experts.

Non-invasive and Cost-Effective Procedures

There are several advantages to non-invasive diagnostic techniques, including liquid biopsies and blood-based biomarkers. These techniques are less intrusive than more established ones like PET scans or lumbar punctures, lessening the patient's danger and pain [39]. Additionally, liquid biopsies and blood-based biomarkers are more affordable and practical, enabling a larger population to receive early Alzheimer's diagnosis.

Facilitating Early Intervention and Treatment

With the rising idea of precision medicine, the availability of new diagnostic technologies allows early intervention and treatment techniques. Early detection enables medical professionals to customise therapies to specific patients depending on each patient's illness stage and features [3]. This personalised strategy improves the efficacy of disease-modifying treatments and raises the chance of successful treatment results.

A comparison of Alzheimer's disease diagnostic tools is summarised in Table 1 [10,18,32,37,44,46-54].

**Table 1:** Comparison of Alzheimer's disease diagnostic tools PET: positron emission tomography; CSF: cerebrospinal fluid. The table has been created by the authors.

Diagnostic tool	Type	Traditional/novel	Sensitivity range	Specificity range	Advantages	Challenges
Brain imaging techniques	Neuroimaging	Traditional	PET (amyloid): 70-90%, MRI: 85%	PET (amyloid): 90-95%, MRI: varied	Molecular insights	High cost, radiation exposure
Cognitive assessment	Clinical	Traditional	60-85%	70-85%	Patient history insights	Subjectivity, cultural bias
CSF analysis	Biomarkers	Novel	85-90%	90-95%	Disease-specific	Invasive, discomfort
Machine learning	Algorithm-based	Novel	80-90%	85-90%	Data pattern recognition	Data quality, interpretability
Blood-based markers	Biomarkers	Novel	70-80%	80-90%	Non-invasive early stage diagnosis	Validation, variability

Challenges and future directions

Standardisation and Validation of Diagnostic Tests

New diagnostic procedures for Alzheimer's disease have difficulties with standardisation and validation. As more and more biomarkers, imaging methods, and machine learning algorithms are researched, it becomes increasingly important to guarantee accurate findings in various contexts. The widespread clinical application of these techniques is hampered by the absence of standardised methods for sample collection, processing, and interpretation [54]. Another difficulty is confirming the validity of diagnostic tests' clinical value and accuracy. The sensitivity, specificity, and predictive usefulness of new instruments must be established through extensive longitudinal research. Developing a solid evidence basis also depends on harmonising findings from research teams and clinical locations [55].

Accessibility and Affordability

While modern diagnostic technologies show promise, it is essential to ensure they are affordable and accessible to various people. Advanced neuroimaging methods, such as targeted PET scans, can be costly and require specialised equipment and skills [56]. Although liquid biopsies and blood-based biomarkers are more widely available options, their use necessitates the creation of accurate, affordable assays that can be quickly incorporated into standard clinical practice. For these innovative procedures to be widely used, insurance coverage and reimbursement challenges must be addressed [57].

Integration of Multiple Diagnostic Approaches

Integrating various methodologies brings possibilities and problems as diagnostic tools develop. Integrating biomarkers, neuroimaging, and machine learning algorithms can improve diagnostic precision, but it is crucial to consider the complexities of interpreting multimodal data [58]. Creating algorithms that combine data from diverse sources and yield therapeutically beneficial findings is challenging. To achieve consistent and understandable results across research and clinical contexts, standardising techniques for integrating various diagnostic modalities is crucial [59].

## Conclusions

Advanced diagnostic methods are essential for the early diagnosis of Alzheimer's disease. These methods, which combine machine learning, neuroimaging, and biomarkers, enable the detection of minor brain alterations even before symptoms appear. Early illness stages offer a critical opportunity for therapies that may slow the course of the disease. These methods improve diagnostic objectivity and accuracy while introducing more affordable and accessible choices, including blood-based biomarkers and liquid biopsies. The cooperation between researchers, physicians, and policymakers promises a bright future even though issues with standardisation, accessibility, ethics, and data integration still exist. As science and technology develop, the road to a better Alzheimer's diagnosis becomes clearer, giving patients and their families fresh hope as they deal with the difficulties of this illness.
